# PI3K/AKT/mTOR/p70S6K Pathway Is Involved in A*β*25-35-Induced Autophagy

**DOI:** 10.1155/2015/161020

**Published:** 2015-10-25

**Authors:** Shengnuo Fan, Bei Zhang, Ping Luan, Beibei Gu, Qing Wan, Xiaoyun Huang, Wang Liao, Jun Liu

**Affiliations:** ^1^Department of Neurology, Sun Yat-sen Memorial Hospital, Sun Yat-sen University, Guangzhou 510120, China; ^2^Department of Neurology, The First Affiliated Hospital, Guangdong Pharmaceutical University, Guangzhou 510080, China; ^3^Medical School, Shenzhen University, Shenzhen, Guangdong 518060, China; ^4^Department of Anesthesiology, Sun Yat-sen Memorial Hospital, Sun Yat-sen University, Guangzhou 510120, China; ^5^Department of Rehabilitation Medicine, Sun Yat-sen Memorial Hospital, Sun Yat-sen University, Guangzhou 510120, China; ^6^Department of Neurology, Houjie Hospital, Dongguan 511711, China

## Abstract

Disruption or deregulation of the autophagy system has been implicated in neurodegenerative disorders such as Alzheimer's disease (AD). A*β* plays an important role in this autophagic system. In many cases, autophagy is regulated by the phosphatidylinositol 3-phosphate kinase/AKT/mammalian target of rapamycin/p70 ribosomal protein S6 kinase (PI3K/AKT/mTOR/p70S6K) signaling pathway. However, whether this signaling pathway is involved in A*β*-induced autophagy in neuronal cells is not known. Here, we studied whether A*β*25-35 induces autophagy in HT22 cells and C57 mice and investigated whether PI3K is involved in the autophagy induction. We found that A*β*25-35 inhibited HT22 cell viability in a dose- and time-dependent manner. A*β*25-35 induced autophagosome formation, the conversion of microtubule-associated protein light chain 3 (LC3), and the suppression of the mTOR pathway both in vitro and in vivo. Furthermore, A*β*25-35 impaired the learning abilities of C57 mice. Our study suggests that A*β*25-35 induces autophagy and the PI3K/AKT/mTOR/p70S6K pathway is involved in the process, which improves our understanding of the pathogenesis of AD and provides an additional model for AD research.

## 1. Introduction

Macroautophagy (autophagy) is a phylogenetically conserved activity that occurs in organisms from yeast to mammals in which damaged organelles and misfolded proteins are degraded and recycled for the maintenance of a healthy cellular environment [1]. In addition to its importance in maintaining cell homeostasis by clearing damaged organelles and waste proteins, autophagy plays roles in many pathological processes, including the degradation of aggregating proteins in neurodegenerative diseases. Disruption or deregulation of the autophagy system has been implicated in neurodegenerative disorders such as Alzheimer's disease (AD) [2].

AD is a devastating disorder that leads to cognitive, behavioral, and memory deficits. The hallmarks of AD are the superfluous accumulation of beta-amyloid (A*β*) into senile plaques and hyperphosphorylated tau into neurofibrillary tangles and neuronal loss in select brain areas involved in learning and memory. It is generally accepted that the excessive A*β* peptide deposition that leads to synapse and neuronal cell loss is involved in AD pathogenesis [3]. A*β* is cleaved from amyloid-*β* protein precursor (APP) and comprises a set of 39–43-residue polypeptides that exert a range of neurotoxic effects that are considered to be important to the evolution of AD [4]. Emerging evidence has shown that A*β*-induced autophagy plays an important role in AD [5, 6].

In mammalian cells, the phosphatidylinositol 3-phosphate kinase/AKT/mammalian target of rapamycin/p70 ribosomal protein S6 kinase (PI3K/AKT/mTOR/p70S6K) signaling pathway is the primary pathway that regulates autophagy when cells are exposed to certain conditions, such as starvation, oxidative stress, infection, and tumor suppression [7]. However, whether the PI3K/AKT/mTOR/p70S6K signaling pathway is involved in A*β*-induced autophagy in neuronal cells is not yet fully understood.

In the present work, we used immortalized murine hippocampal neurons (HT22 cells), which possess functional cholinergic properties when differentiated [8–10], as a cell model to study whether A*β* exposure induces autophagy. Among all A*β* fragments used in scientific researches, A*β*25-35 is the shortest active fragment that showed similar neurotoxicity effect to a full-length A*β*. Considering A*β*25-35 can be easily synthesized, we choose this fragment as reagent in the study [11]. Additionally, we examined the morphocytological changes in mouse hippocampal cells after the intracerebroventricular administration of A*β*25-35. We further investigated whether the PI3K/AKT/mTOR/p70S6K pathway was involved in these autophagic processes. Our findings provide valuable information about the mechanism of the regulation of autophagy in AD pathogenesis.

## 2. Materials and Methods

### 2.1. Reagents and Antibodies

Dulbecco's modified Eagle's medium (DMEM), fetal bovine serum (FBS), neurobasal medium, and N2 supplement were obtained from Gibco (New York, USA). A*β*25-35 was synthesized by Shanghai Sangon Biological Engineering Technology & Services Co., Ltd. A cell counting kit-8 (CCK-8) was acquired from Dojin Laboratories (Kumamoto, Kyushu, Japan). DAPI was obtained from Invitrogen/Life Technologies (Carlsbad, CA, USA). The antibodies anti-phospho-AKT (Ser473), anti-AKT, anti-p70S6K, anti-p-p70S6K, and anti-tubulin and secondary antibody horseradish peroxidase- (HRP-) conjugated goat-anti-rabbit IgG were obtained from Cell Signaling Technology (USA). Anti-LC3 was acquired from the MBL International Corporation. The Western blot chemiluminescent horseradish peroxidase substrate was purchased from Millipore (USA). All other experimental supplies and reagents were purchased from Invitrogen, Thermo Fisher, and MR Biotech.

### 2.2. Cell Culture, Differentiation, A*β* Preparation, and Treatment

HT22 cells were maintained in DMEM supplemented with 10% FBS as previously described [8, 10] and differentiated in neurobasal medium containing 1 × N2 supplement for 24–48 h before use [9]. A*β*25-35 was diluted in sterile saline at a concentration of 0.5 mM and was maintained at 37°C for 7 days to pre-age the peptide [11]. The aged A*β* solution was diluted to different concentrations before treatment. Bafilomycin A1 (100 nM) and rapamycin (1 *μ*M) were used accordingly. Different concentrations of A*β*25-35 were added to the HT22 cells for various times.

### 2.3. Cell Viability Assay

The viabilities of the HT22 cells were evaluated by CCK-8. Briefly, after the treatment of the different groups, 10 *μ*L/well of CCK-8 reagent was added to the cells, which were then incubated for 1.5 h at 37°C with 5% CO_2_ in dark conditions. The optical density (OD) was measured at an absorbance wavelength of 450 nm with a multifunctional microplate reader (SpectraMax M5, USA).

### 2.4. Immunofluorescence Assay

The appropriate concentrations (10 and 40 *μ*M) of A*β* as determined with the CCK-8 test were used in the cultured HT22 cells for 24 h with or without bafilomycin A1. Rapamycin (1 *μ*M) was chosen as a positive control. The different groups were fixed in 4% paraformaldehyde for 15 min at room temperature and then permeabilized with 0.25% triton X-100 for 10 min. After blocking with 1% bovine serum albumin in PBS for 30 min, the samples were incubated with anti-LC3 (1 : 500) overnight at 4°C in a humidified chamber. After washing three times in PBS, incubating for 60 min with HRP-conjugated goat-anti-rabbit IgG, and repeating the three washes, the samples were stained with DAPI for 5 min. Images were acquired with an upright immunofluorescence microscope (BX51WI, Olympus, USA).

### 2.5. Mice and A*β* Intracranial Injection

All experiments were carried out in accordance with guidelines approved by ethical committee of Sun Yat-sen University, which includes minimizing the number of animals used and their suffering.

A total of 24 male 6-month-old C57BL/6J mice weighing 28.1 ± 1.4 g were used in this study. There were no significant changes in body weight between or within the groups of mice. The 24 mice were randomly divided into three groups of 8 mice each that were treated with sterile saline, low-dose A*β*, and high-dose A*β* group. The animal care and the experimental procedures of this study were approved by the Animal Care and Ethics Committee at Sun Yat-sen University, China.

The pre-aged A*β*25-35 was diluted to 2 *μ*g/*μ*L with sterile saline before injection. A*β*25-35 was administered intracerebroventricularly (i.c.v.). Immediately before surgery, the mice were weighed and then anaesthetized with 10% chloral hydrate. A stereotaxic apparatus (Wood Dale, IL, USA) was used. Burr holes were made to access the hippocampus using the following previously determined coordinates relative to the bregma: anteroposterior, −1.5 mm; lateral, −1.0 mm; and vertical, 2.0 mm. The injections were performed with a Hamilton microsyringe equipped with a 3 mm needle. The injections were administered as follows: Group A received sterile saline (0.6 *μ*L); Group B received low-dose A*β*25-35 (0.3 *μ*L); and Group C received high-dose A*β*25-35 (0.6 *μ*L). The injection time was 5 min, and the needle was maintained at the injection site for 2 min prior to slow withdrawal.

### 2.6. Behavioral Testing

Spatial learning and memory functions were assessed with the Morris Water Maze (MWM) 2 weeks after the injections. The procedure consisted of 1 day of adaptation tests without a platform and 5 days of hidden platform tests plus a spatial probe test that was performed 24 h after the last hidden platform test. For each trial, the mouse was allowed to swim to find the hidden platform for a maximum of 60 s. The animals that did not find the platform within this time limit were guided to the platform and kept there for 15 s. The animals were given 4 trials per day. The intertrial interval was 15 min. The distal start positions were semirandomly selected. The time required to find and climb onto the platform was recorded as the latency. The probe trial was performed 24 h after the last acquisition test. During the probe trials, the platform was removed, and the mice were free to swim in the water for 60 s. The numbers of crossings of the platform location and the time spent in the target quadrant were measured to assess the acquisition measurement of the water maze task.

### 2.7. Transmission Electron Microscopy (TEM)

Upon the completion of behavioral testing, the mice were perfused with 4% paraformaldehyde. The brains were rapidly removed and fixed in ice-cold glutaraldehyde (3% in 0.1 M PBS, pH 7.4) for 1 h. The hippocampal samples were cut into 2 mm^3^ pieces and postfixed 1% osmium tetroxide in PBS for 1 h at 4°C, processed through a graded series of acetone, embedded in Araldite, and polymerized overnight at 60°C. Thin sections (60 nm) were collected on formvar-coated, single-slot grids, stained with uranyl acetate and lead citrate, and then viewed on a FEI Tecnai G2 Spirit TWIN transmission electron microscope (OR, USA).

### 2.8. Western Blot Analysis

The HT22 cells were exposed to various conditions for various durations and were then washed gently with PBS twice and lysed with RIPA lysis buffer. Hippocampal samples from the mice in each group were collected after the completion of the behavioral testing. The tissues were homogenized using 10 up-and-down strokes of a prechilled Teflon-glass homogenizer in lysis buffer. The lysates were then sonicated and centrifuged. Subsequent to boiling and denaturing, the cell and tissue protein samples (30 *μ*g) were subjected to 12% SDS-polyacrylamide gel electrophoresis and transferred to a PVDF membrane (Millipore, USA). After blocking with 5% nonfat milk, the membranes were incubated with the following primary antibodies overnight at 4°C: anti-phospho-AKT (1 : 1,000), anti-AKT (1 : 1,000), anti-phospho-p70S6K (1 : 1,000), anti-p70S6K (1 : 1,000), anti-LC3 (1 : 1,000), and anti-Tublin (1 : 2,000). After washing with TBST three times, the membranes were incubated with anti-rabbit secondary antibodies (dilution, 1 : 2,000) for 60 min. The washings were then repeated. The membranes were incubated with horseradish peroxidase substrate for 5 min, and the fluorescence bands were detected with X-ray films. The intensities of the bands were quantified with a Gel-Pro Analyzer (Media Cybernetics Inc., USA).

### 2.9. Statistical Analyses

SPSS 13.0 software (SPSS Inc., Chicago, IL, USA) was used for the data processing. All of the data are expressed as the mean ± the standard error (SE). One-way ANOVAs with post hoc tests or* t*-tests were used for the statistical analyses. *P* < 0.05 was required for results to be considered statistically significant.

## 3. Results

### 3.1. A*β*25-35 Decreased Cell Viability in a Dose- and Time-Dependent Manner

**Figure 1:**
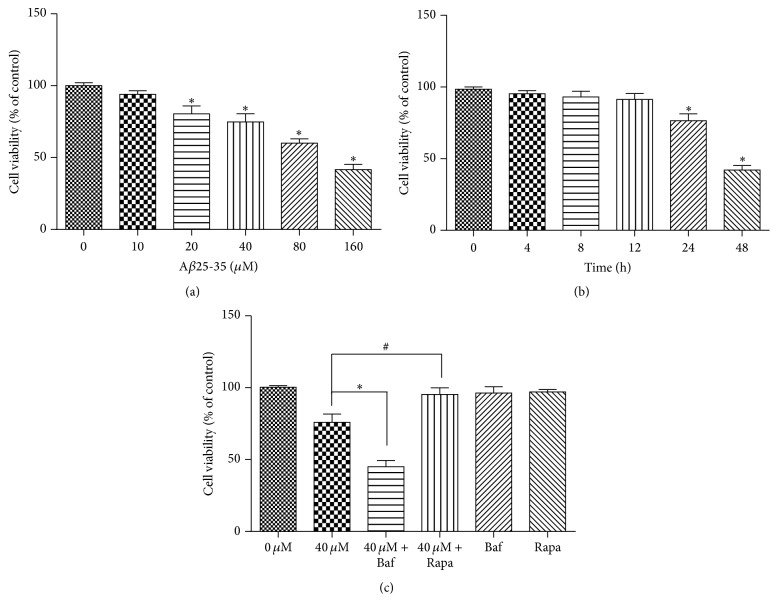
A*β*25-35 inhibited the growth of HT22 cells. The viabilities of the HT22 cells incubated with (a) various doses of A*β*25-35 for 24 h, (b) 40 *μ*M A*β*25-35 for various times, and (c) 40 *μ*M A*β*25-35 with bafilomycin A1 or rapamycin were evaluated with CCK8 assays. ^*∗*#^ *P* < 0.05.

When the HT22 cells were treated with increasing doses of A*β*25-35 for 24 h, the viabilities of the HT22 cells decreased. As shown in Figure 1, A*β*25-35 significantly inhibited the growth of HT22 cells at doses greater than or equal to 40 *μ*M (*P* < 0.05), and 40 *μ*M A*β*25-35 resulted in remarkably increased cytotoxicity in the HT22 cells at various times ranging from 24 h to 48 h (*P* < 0.05). The autophagy inhibitor bafilomycin A1 significantly exacerbated A*β*25-35 cytotoxicity, and the autophagy activator rapamycin had the opposite effect. Neither bafilomycin A1 nor rapamycin exhibited significant toxicity to the HT22 cells when applied alone. We selected 40 *μ*M as the optimal concentration of A*β*25-35 for the subsequent experiments.

### 3.2. A*β*25-35 Induced Autophagosome Formation

**Figure 2:**
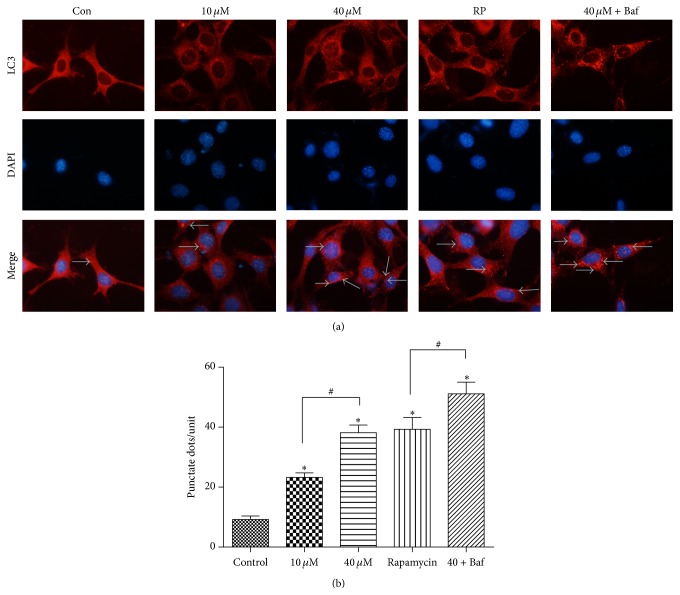
LC3 immunoreactivity is shown. HT22 cells were stained for LC3 (red arrows) and with the nuclear dye DAPI (blue) following the different treatments for 24 h. (Control) Negative control group: LC3 punctate dots (red) can barely be detected. (10 *μ*M) 10 *μ*M A*β*25-35 group: the dots exhibited a scattered distribution in the perinuclear region. (40 *μ*M) 40 *μ*M A*β*25-35 group: the dots are evenly spread around the perinuclear and cytoplasm regions. (RP) rapamycin group. (40 *μ*M + Baf) 40 *μ*M A*β*25-35 and bafilomycin A1 (Baf, 100 nM) group: the cells are filled with cluster-like dots. ^*∗*#^ *P* < 0.05.

Punctate LC3 staining was observed in the perinuclear regions in the various groups. After A*β*25-35 treatment for 24 h, LC3 immunostaining was evaluated with an immunofluorescence microscope, and the presence of autophagosomes was detected by the visualization of punctate dots. As shown in Figure 2, 40 *μ*M A*β*25-35 induced a greater number of punctate dots than 10 *μ*M. The combination of A*β*25-35 and bafilomycin A1 produced more dots than A*β*25-35 alone. Rapamycin (RP, 1 *μ*M) served as a positive control.

### 3.3. Intracranial Injection of A*β*25-35 Impaired the Learning Abilities of C57 Mice

**Figure 3:**
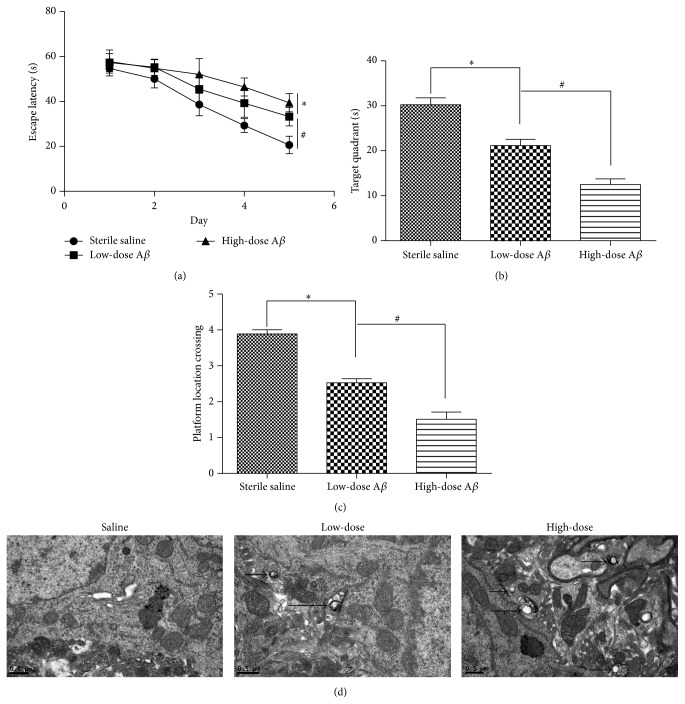
Intracranial injection of A*β*25-35 impaired the learning abilities of C57 mice in the MWM. (a) The mean escape latencies of the mice in the place navigation test. (b) The numbers of platform area crossings in the probe trials. (c) The times spent in the target quadrant in the probe trials. (d) Representative ultrastructural appearance of autophagic vacuoles (AVs, arrows) in mice hippocampus of each group. As shown in TEM pictures of saline group, there was barely any accumulation of autophagic vesicle in axons. Low-dose A*β*25-35 group showed significant accumulation of AVs, while high-dose A*β*25-35 group depicted more AVs than low-dose group. ^*∗*#^ *P* < 0.05.

Two weeks after the intracranial injections of A*β*25-35, the MWM test was conducted to evaluate the learning and memory abilities of the mice. In the hidden-platform tests, the latencies diminished across training days in all the three groups. As shown in Figure 3, saline group exhibited lower escape latencies (Figure 3(a)) than A*β*25-35 groups across the successive days of training. Compared to low-dose group, high-dose group behaved worse, which indicated a dose-dependent effect of A*β*25-35. Probe trials were performed 24 h after the final place navigation tests. The numbers of platform area crossings and times spent in the target quadrant during the 60 s trials (Figures 3(b) and 3(c)) revealed that saline group performed significantly better than A*β*25-35 groups. Dose-dependent effects were also manifested in these measurements.

### 3.4. Intracranial Injection of A*β*25-35 Induced a Large Accumulation of Autophagic Vesicles (AVs)

TEM revealed a large accumulation of autophagic vesicles (AVs) in the hippocampus 2 weeks after the injection of A*β*25-35. As depicted in Figure 3(d), there was extensive accumulation of AVs in the axons of A*β*25-35 groups. Compared to low-dose group, high-dose group exhibited a greater number of AVs, which indicated a dose-dependent effect. In contrast, autophagosomes were rarely observed in the neurons of saline group.

### 3.5. PI3K/AKT/mTOR/p70S6K Pathway Is Involved in A*β*25-35-Induced Autophagy

**Figure 4:**
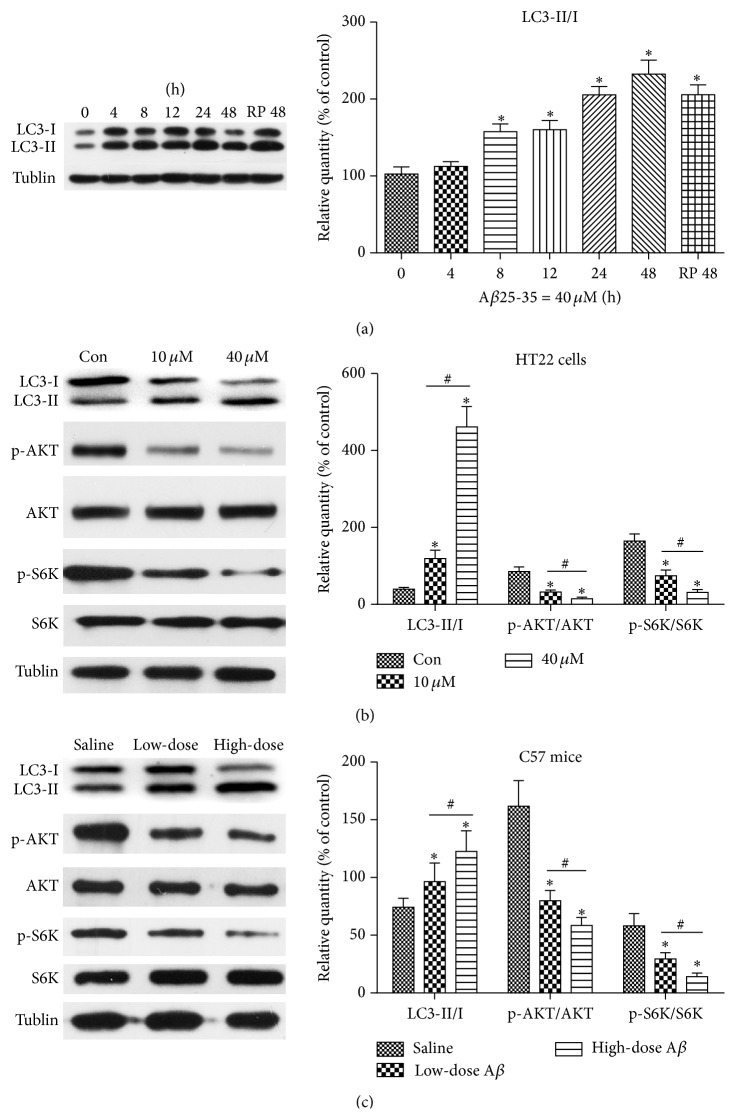
The effects of A*β*25-35 treatment on the LC3 II/I ratio and the levels of phosphorylation of AKT and p70S6K (S6K) in HT22 cells and C57 mice. (a) HT22 cells were treated with 40 *μ*M A*β*25-35 for various times ranging from 0 h to 48 h. Rapamycin (1 *μ*M, 48 h) served as a positive control. (b) The HT22 cells were divided into an untreated group, a 10 *μ*M A*β*25-35 group, and a 40 *μ*M A*β*25-35 group and treated for 24 h. The column graphs below the panels depict the relative expressions of LC3 II/I and phosphorylated AKT and p70S6K (S6K). (c) The mice were intracerebroventricularly administered sterile saline, low-dose A*β*25-35 (0.3 *μ*L), or high-dose A*β*25-35 (0.6 *μ*L). The expressions of Tublin confirmed equal protein loading. ^*∗*#^ *P* < 0.05.

In vitro, we first detected the protein expressions of microtubule-associated protein light chain 3 in the HT22 cells (LC3) following A*β*25-35 (40 *μ*M) treatment for various times and following A*β*25-35 treatment at different concentrations for 24 h. Consequently (Figure 4(a)), the conversion of the LC3 soluble form (LC3-I) into the autophagosome-associated form (LC3-II) (i.e., the LC3-II/I ratio) increased as the treatment time increased from 0 h to 48 h. The 40 *μ*M A*β*25-35 treatment resulted in a greater increase in this ratio than the 10 *μ*M A*β*25-35 treatment (Figure 4(b)). We next examined whether the PI3K/AKT/mTOR/p70S6K pathway was involved in this process by analyzing the levels of phosphorylated p70S6K and AKT (Figure 4(b)). We found that A*β*25-35 remarkably decreased the levels of phosphorylated p70S6K and AKT compared to the nontreated group.

In vivo, an augmentation of the LC3-II/I ratio and reductions in p70S6K and AKT were also detected following the injections of A*β*25-35 (Figure 4(c)).

Together, our results indicated that A*β*25-35 treatment inhibited HT22 cell viability in a dose- and time-dependent manner and dose-dependently impaired the learning abilities of the C57 mice. A*β*25-35 treatment induced autophagy and PI3K/AKT/mTOR/p70S6K pathway is involved in this autophagic activity.

## 4. Discussion

According to the amyloid cascade hypothesis, A*β* is a major etiological agent that causes devastating neurotoxicity, including oxidative stress, unbalanced calcium levels, neurofibrillary tangles, inflammatory reactions, synaptic dysfunction, and hippocampal neuron loss [12, 13]. Our previous work [14], together with a substantial volume of in vitro and in vivo research from around the world, has verified the toxicity of A*β* [15, 16]. The present study also revealed that A*β*25-35 inhibited HT22 cell viability in a dose- and time-dependent manner. Numerous studies have proven that the amelioration of this neurotoxicity and anti-A*β* strategies improve neuronal survival, spatial memory, synaptic plasticity, and calcium homeostasis in AD models [17–19]. Therefore, the goal of achieving A*β* clearance is of great significance to AD therapy.

Autophagy has been indicated to play an important role in the pathogenesis of AD [2, 20], which occurs extensively in transgenic mouse models that overexpress A*β* [21] and in vitro models [22, 23]. In our work, we successfully induced autophagy via the treatment of HT22 cells and C57 mice with A*β*25-35. Using generally accepted markers of autophagy [24], that is, LC3 punctate dot immunofluorescent, AV detection via TEM, and the LC3 II/I ratio, our research identified the formation of autophagosomes. Autophagy is recognized as a double-edged sword in the regulation of health and disease [20]. Emerging evidence shows that hyperactive autophagy is harmful to neuron survival. The inhibition of certain autophagic activities might prevent neurite degeneration [25]. Interestingly, regarding AD research, autophagy has been proven to be beneficial in most cases [16, 26, 27]. Hence, we presume that the autophagy induced by A*β*25-35 might act as a self-defense mechanism via the intracellular clearance of peptide deposits and the cellular organelles that are damaged by neurotoxicity. The upregulation of autophagy might protect against the neuronal degeneration that is induced by A*β*25-35 exposure. Recent studies have shown that enhancing autophagy with rapamycin rescues AD-like pathology and cognitive deficits in murine AD models [26, 27] and that blocking autophagy aggravates neurite degeneration [28]. The results of these studies are consistent with our findings. However, sufficient and appropriate autophagy is required for proper function because pathologic autophagy also occurs. Compared to nondemented groups, heavy accumulations of autophagosomes are present in the AD cortex and hippocampus. The accumulation of autophagosomes in AD might be due to the stimulation of autophagy or a residually slow rate of autophagosome formation combined with a failure to complete sufficient lysosomal fusion and digestion [29]. Thus, there should be an equilibrium point in the modulation of autophagy. Recent research has indicated that primary lysosomal dysfunction causes cargo-specific deficits in axonal transport that lead to Alzheimer's-like neuritic dystrophy [30]. Therefore, it is possible that the deposition of A*β* leads to a dysfunction of lysosomal proteolysis that would account for the accumulation of autophagic structures. However, further study is needed to understand this autophagic system.

The function of mTOR is primarily mediated by mTOR complex 1 (mTORC1) and mTORC2. mTORC1 is a major negative regulator of autophagy and is also a key homeostatic regulator of cell growth, proliferation, and survival [31]. As the major upstream modulator, the PI3K pathway regulates autophagy by phosphorylating AKT at serine residue 473 (Ser473), which influences the downstream elements p70S6K and 4E-BP1 [32]. The PI3K/AKT/mTOR/p70S6K pathway plays a vital role in the central nervous system (CNS), particularly in synaptic development and function [33, 34]. Dysregulation of the PI3K/AKT/mTOR/p70S6K pathway has commonly been reported in the brains of AD patients, and both hypoactivation and hyperactivation are linked to autophagy disruptions related to the pathology of AD [35, 36]. In our study, we found that A*β*25-35 remarkably decreased the levels of phosphorylated p70S6K and AKT compared to the control group both in vitro and in vivo, which supports the speculation that PI3K pathway is involved in this autophagic process.

## 5. Conclusion 

This study indicates that A*β*25-35 treatment inhibits HT22 cell viability in a dose- and time-dependent manner and dose-dependently impairs the learning abilities of the C57 mice. The study also suggests the PI3K/AKT/mTOR/p70S6K pathway is involved in A*β*25-35-induced autophagy both in HT22 cells and in C57 mice, and these findings might provide a better understanding of AD pathogenesis and an additional model for AD research.
